# Free Base Porphyrins as Ionophores for Heavy Metal Sensors

**DOI:** 10.3390/s8084995

**Published:** 2008-08-25

**Authors:** Dana Vlascici, Eugenia Fagadar Cosma, Elena Maria Pica, Viorica Cosma, Otilia Bizerea, Gheorghe Mihailescu, Liliana Olenic

**Affiliations:** 1 West University of Timisoara, 4 V. Parvan Ave, Timisoara 300223 Timis, Romania; 2 Institute of Chemistry –Timişoara of Romanian Academy, 24 M. Viteazul Ave, 300223-Timisoara,Romania; 3 Technical University of Cluj-Napoca, Faculty of Science and Materials Engineering, 15 Constantin Daicoviciu Street, 400020 Cluj-Napoca, Romania; 4 Nationale Institute of Research and Development for Isotopic and Molecular Technologies, 71-103 Donath Street 400293, Cluj-Napoca, Romania E-Mails: efagadar@yahoo.com (E.F.C.); epica@yahoo.com (E.M.P.); vioricacosma@yahoo.com (V.C.); obizerea@yahoo.com (O.B.); gigim@itim-cj.ro (G.M.); olenic@itim-cj.ro (L.O.)

**Keywords:** Porphyrins, ionophores, sensors, heavy metals, PVC-matrix

## Abstract

Two functionalized porphyrins: 5,10,15,20-tetrakis(3,4-dimethoxyphenyl) porphyrin (A) and 5,10,15,20-tetrakis(3-hydroxyphenyl)porphyrin (B) obtained and characterized by us were used as ionophores (I) for preparing PVC-based membrane sensors selective to Ag^+^, Pb^2+^ and Cu^2+^. The membranes were prepared using three different plasticizers: (bis(2-ethylhexyl)sebacate (DOS), dioctylphtalate (DOP), *o*-nitro-phenyl octyl ether (NPOE) and potassium tetrakis(4-chlorophenyl)borate (KTClPB) as additive. The functional parameters (linear concentration range, slope and selectivity) of the sensors with membrane composition: (I:PVC:KTClPB:Plasticizer) in different ratios were investigated. The best results were obtained for the membranes in the ratio I:PVC:KTClPB:Plasticizer 10:165:5:330. The influence of pH on the sensors response was studied. The sensors were used for a period of four months and their utility has been tested on synthetic and real samples.

## 1. Introduction

Potentiometric determination of ions offers advantages such as high selectivity, sensitivity, good precision, simplicity, portable, non-destructive analysis and low cost. Many porphyrins [1-10] and metalloporphyrins were used in the last years as ionophores because of the importance of developing new polymeric membrane ion-selective electrodes.

A silver-selective electrode based on *meso*-tetratolylporphyrin was reported [1]. The electrode showed a Nernstian answer with a slope of 59.2 mV/decade in a working concentration range of 10^-7^− 10^-1^ M Ag^+^. The response time of the electrode was less than 10 s with a lifetime of 3 months. The electrode was used for the determination of silver in real samples and as an indicator electrode in potentiometric titrations. Determination of silver ion by simple methods is very important in chemical, clinical and environmental analysis due to the increasing use of silver compounds in industry and medicine.

Lead-selective electrodes have been extensively reported because of the toxic effects of lead on human health on one hand, and its increased industrial on the other. Among them, one lead-selective sensor based on porphyrins was reported. The sensor is based on *meso*-tetraphenylporphyrin as ionophore and has a working concentration range between 10^-5^−10^-2^ M Pb^2+^ with a detection limit of 8.5×10^-6^ M. The response time was 15 s and the lifetime 3 months. The sensor works in a pH range from 5-7.5 with good selectivity coefficients and was used as an indicator electrode in the potentiometric titration of the lead ion [2].

Copper has a vital importance in many biological systems and it is used for many industrial, agricultural and domestic purposes. It is toxic at high level concentrations so its determination in medicinal, environmental and industrial samples is very important. One copper-selective sensor based on *meso*-tetrakis-[4-(diallylmethylsilyl)phenyl]porphyrin was reported previously [3]. The sensor had a working concentration range between 4.4×10^-6^–1.0×10^-1^ M with a Nernstian slope of 29.3 mV/decade of activity in a pH range 2.8-7.9. The response time was about 8 s with a 4 months lifetime. The utility of the sensor was demonstrated by determining copper in vegetable foliage and swimming pool water samples. Other porphyrin-based sensors for Ni^2+^ and Zn^2+^ determination have also been reported [4-10].

In connection with our previous concerns about the use of porphyrins in the construction of ion selective electrodes [11, 12], in the present paper, two functionalized porphyrins: 5,10,15,20-tetrakis-(3,4-dimethoxyphenyl) porphyrin (**A**) and 5,10,15,20-tetrakis(3-hydroxyphenyl)porphyrin (**B**) synthesized and characterized by us as reported earlier [13] were tested as ionophores for cation-selective electrodes in polymeric matrix. Different membrane compositions were made by varying the amount of ionophore, additive and by using three different plasticizers: (bis(2-ethylhexyl)-sebacate(DOS), dioctylphtalate (DOP) and *o*-nitrophenyl octyl ether (NPOE). The best results were obtained for the membranes in the ratio I:PVC:KTClPB:Plasticizer 10:165:5:330. The sensitivity, selectivity, pH influence and effect of the plasticizer on the behavior of the sensors were studied and the obtained results are presented below.

The sensors were used for a period of four months and their utility has been tested in synthetic and real samples.

## 2. Results and Discussion

### 2.1. Response characteristics of the electrodes

**Figure 1.**
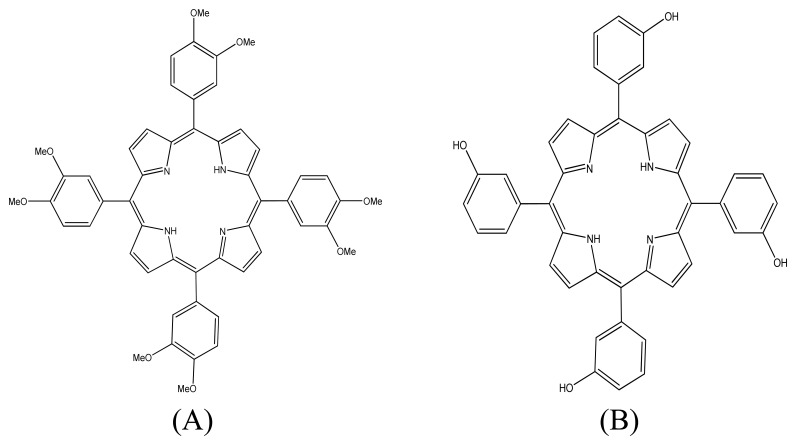
The structures of free base porphyrins 5,10,15,20-tetrakis(3,4-dimetoxyphenyl) porphyrin (**A**) and 5,10,15,20-tetrakis (3-hydroxyphenyl) porphyrin (**B**) used as ionophores.

The potentiometric answer of each ISE depends not only on the nature of the ionophore used (in our case porphyrins **A** and **B** shown in Figure 1), but also significantly on the nature and amount of plasticizers and additives used.

Because of these, the influence of membrane compositions having different amounts of ionophore, additive and plasticizers like (bis(2-ethylhexyl)sebacate(DOS), dioctylphtalate (DOP) and *o*-nitro-phenyl octyl ether (NPOE) on the potentiometric response was investigated. The obtained sensors were tested in solutions from 1×10^-6^−1×10^-1^ M of the following cations: Ag^+^, K^+^, Na^+^, Ni^2+^, Cu^2+^, Cd^2+^, Zn^2+^, Mg^2+^ and Pb^2+^. Potassium tetrakis(4-chlorophenyl)borate (KTClPB) was used as additive to all prepared membranes.

**Figure 2.**
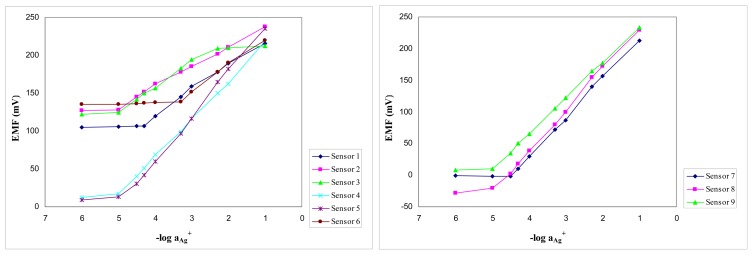
Potentiometric response of the sensors 1-6 based on ionophore **A** and 7-9 based on ionophore **B** toward Ag^+^.

It was found that the potentiometric answer toward silver of all the obtained sensors corresponds to the cation lipophilicity sequence (Hofmeister series of cations) [14]:

$${\text{Ag}}^{+}>{\text{K}}^{+}>{\text{NH}}_{4}^{+}>{\text{Na}}^{+}>{\text{Li}}^{+}>{\text{Ca}}^{2+}>{\text{Pb}}^{2+}>{\text{Cu}}^{2+}$$

and it is presented in Table 1 and Figure 2. A sensor with a blank membrane having the composition PVC:KTClPB:Plasticizer = 165:5:330 showed a working concentration range for silver from 10^-3^ to 10^-1^ M with a sub-Nernstian slope of (20-24) mV/decade. This is the reason why the obtained results are ascribed to the porphyrins **A** and **B** used as ionophores.

The response toward silver was sub-Nernstian for the electrodes 1-3 and 6, but the increase of the ionophore and additive amount in the membrane composition conducts to electrodes having slopes closer to theoretical value and good working concentration range. The best results were obtained for sensor no. 5, based on porphyrin **A** and having NPOE as a plasticizer in the membrane, which has a working concentration range of 1×10^-5^−1×10^-1^ M and a slope of 57.9 mV/decade of activity and for sensor no. 8, based on porphyrin **B** and plasticized with DOS, with a working concentration range from 8×10^-6^−1×10^-1^ M and a slope of 64.5 mV/decade of activity.

### 2.2. Potentiometric selectivity

**Figure 3.**
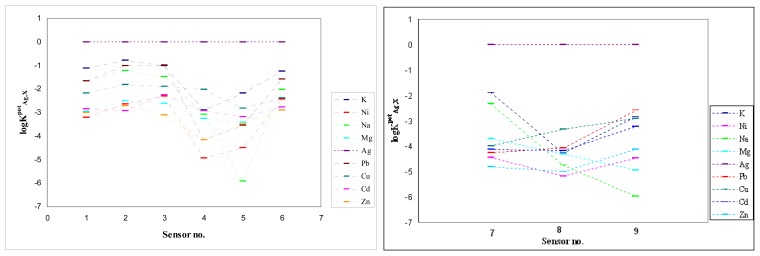
Selectivity coefficients of the sensors 1-6 based on ionophore A and 7-9 based on ionophore B calculated by separate solution method (SSM).

Selectivity is perhaps the most important characteristic of any sensor which defines the extent to which it may be employed in the determination of a particular ion in the presence of the other interfering ions. The selectivity coefficients, ${\log\text{K}}_{\text{Ag},\text{X}}^{\text{pot}}$, were calculated by separate solution method (SSM) [15] and are comparatively presented in figure 3.

Analyzing the values of the selectivity coefficients it results that all the obtained sensors are silver-selective, the best results being obtained again for sensors no. 5 and 8 which have very good selectivity for silver over all the other tested cations.

From Figure 3 it can be seen that sensors no. 4 and 9, with the exception of silver, do not respect the Hofmeister sequence for cations. For sensor no. 4 the sequence is:

$${\text{Ag}}^{+}>{\text{Cu}}^{2+}>{\text{K}}^{+}>{\text{Cd}}^{2+}{>\text{Na}}^{+}>{\text{Mg}}^{2+}>{\text{Pb}}^{2+}>{\text{Zn}}^{2+}>{\text{Ni}}^{2+}$$

**Figure 4.**
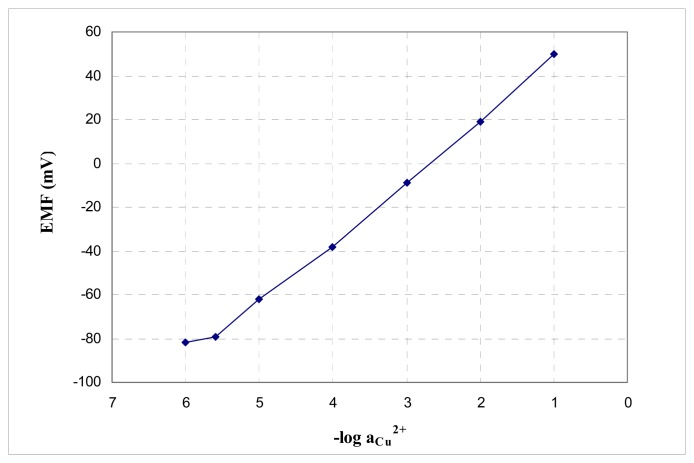
Potentiometric response of the sensor no. 4 based on ionophore **A** toward Cu^2+^.

So, in the absence of silver, the sensor no. 4 based on porphyrin **A** plasticized with DOS could be used as a copper-selective electrode, the answer to copper being presented in Figure 4.

The sensor has a working concentration range of 2.5×10^-6^ M−1×10^-1^ M, a slope of 27.8 mV/decade copper, a detection limit of 2×10^-6^ M and a moderate selectivity over the other tested cations (with the exception of silver which interferes totally).

Sensor no. 9, based on porphyrin **B** plasticized with NPOE, could be used, in the absence of silver, as a lead-selective sensor, having the sequence of cations:

$${\text{Ag}}^{+}>{\text{Pb}}^{2+}>{\text{K}}^{+}>{\text{Cu}}^{2+}>{\text{Cd}}^{2+}>{\text{Zn}}^{2+}>{\text{Ni}}^{2+}>{\text{Mg}}^{2+}>{\text{Na}}^{+}$$

**Figure 5.**
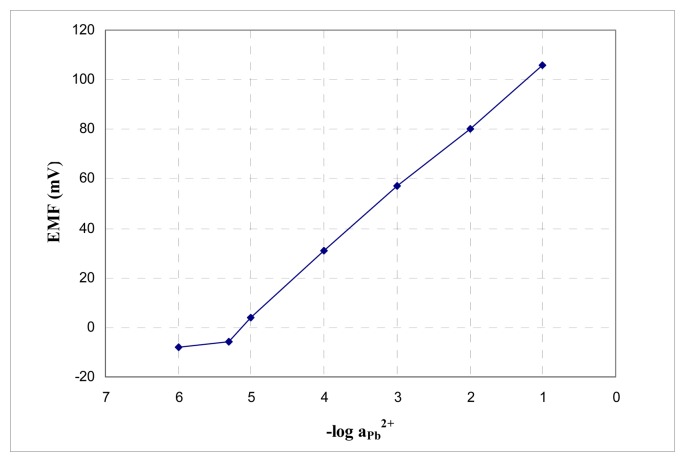
Potentiometric response of the sensor no. 9 based on ionophore **B** toward Pb^2+^.

The sensor could be used in the range 5×10^-6^−1×10^-1^ M with a slope of 25.8 mV/decade of activity lead. The potentiometric answer of the sensor is presented in Figure 5.

The reproductibility of the sensors was determined by a repeated monitoring of potentials (15 measurements) on the same portion of sample. The standard deviation obtained for sensors no. 8, 4 and 9 was ± 1.8 mV, ± 1.5 mV respectively ± 1.2 mV.

### 2.3. Effect of the pH

**Figure 6.**
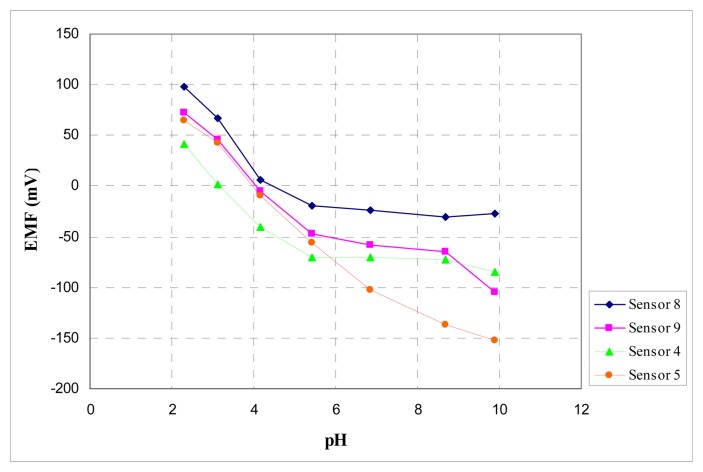
Effect of the pH of the test solution on the potential response of the sensors with best potentiometric answers.

The influence of the pH of the test solutions on the potential response of the electrodes was studied in the pH range 2.0-10.0 in the 0.01M NaCl solution (adjusted with HNO_3_ and NaOH). The results are given in the figure 6.

All membranes plasticized with DOS and NPOE displays same pH sensitivity in acidic solutions probably because of the partial protonation of porphyrins. The potentiometric response of the sensors no. 8 and 9 based on tetra-(3-hydroxyphenyl)porphyrin plasticized with DOS and NPOE are independent of pH in the pH range 5-10 and 5-9 respectively. The response of the membranes with ionophore **A**, tetra-(3,4-dimethoxyphenyl) porphyrin and DOS are independent of pH in the range 5-10. The sensor no. 5, based on the same porphyrin with NPOE, displays a pH response with a slope of about 30 mV/decade and it was not used for analytical applications.

### 2.4. Analytical applications

#### Silver-selective sensor

The silver-selective sensor (sensor no. 8) was used to determine chloride from meat products by potentiometric titration. Volhard titration adopted as reference method was carried to compare the results obtained for chloride potentiometric determination. For each type of meat product the methods were applied on four samples. The obtained results are presented in Table 2:

#### Lead-selective sensor

The practical applicability of the Pb^2+^-selective sensor (sensor no. 9) was made to determine lead from synthetic and real samples. For the potentiometric determination of lead measurements were carried out by standard addition method. The results were compared with those obtained by atomic absorption spectrometry (AAS) and are presented in Table 3.

The results obtained by potentiometry were in good agreement with those obtained by AAS. The recovery tests for synthetic samples, by potentiometry with the sensor, are situated in the range 99.1-101.6 %.

#### Copper-selective sensor

The copper-selective sensor (sensor no. 4) was used to determine Cu^2+^ from synthetic samples by direct potentiometry and by standard addition method. The obtained results are presented in Table 4 comparative to those obtained by AAS.

## 3. Conclusions

Two functionalized porphyrins were tested as ionophores in polymeric membrane cation-selective electrodes. The best results as silver-selective electrodes were obtained for the membrane based on tetra-(3-hydroxyphenyl) porphyrin (**B**) plasticized with DOS which has a working concentration range from 8×10^-6^−1×10^-1^ M, a slope of (64.5±1.8) mV/decade of activity and very good values of the selectivity coefficients. The detection limit of the sensor is 7.0×10^-6^ M. The useful pH-range is 5-10. The sensor was used for the determination of chlorides in meat by potentiometric titration.

In the absence of silver, the sensor having a membrane based on tetra-(3,4-dimetoxyphenyl) porphyrin (A) plasticized with DOS could be used in a pH range from 5-10 as a copper-selective electrode with a working concentration range of 2.5×10^-6^ M−1×10^-1^ M, a slope of (27.8±1.5) mV/decade copper, a detection limit of 2×10^-6^ M and a moderate selectivity over the other tested cations. The sensor was used for the determination of copper from synthetic samples.

The sensor based on tetra-(3-hydroxyphenyl) porphyrin with NPOE as plasticizer could be used in a pH range from 5-9, in the absence of silver which interferes, as a lead-selective sensor. It has a linear range from 5×10^-6^−1×10^-1^ M with a slope of (25.8±1.0) mV/decade of activity lead and a detection limit of 3×10^-6^ M. The sensor was used for the determination of lead from real and synthetic samples.

All the sensors were used for a period of 4 months.

## 4. Experimental Section

### 4.1. Reagents

The porphyrins: tetra-(3,4-dimetoxyphenyl) porphyrin (**A**) and tetra-(3-hydroxyphenyl) porphyrin (**B**) were synthesized, purified and characterized by HPLC, TLC, UV-vis, fluorescence, MS, ^1^H-NMR and ^13^C-NMR analysis, in accordance with previously published procedures [13]. For membrane preparation, poly(vinyl)chloride (PVC) high molecular weight, bis(2-ethylhexyl)sebacate (DOS), *o*-nitrophenyl octylether (NPOE), dioctylphtalate (DOP), potassium tetrakis(4-chlorophenyl)borate (KTClPB) and tetrahydrofuran (THF) were purchased from Fluka and Merck. All salts, acids and base were of analytical reagent grade. Double distilled water was used. The performance of each sensor was investigated by measuring its potential in the concentration range 10^-6^-10^-1^ M of different cationic solutions. 0.1 M stock solutions were prepared by dissolving metal nitrates in double distilled water and standardized if necessary. All working solutions were prepared by gradual dilution of the stock solutions.

### 4.2. Electrode preparation and measurements

A mixture (250 mg) of PVC, plasticizer, ionophore (porphyrins) and the ionic additive, in different ratios were mixed in THF (10 mL) and was intensively stirred until all components were dissolved. The homogeneous liquid was poured on the conductive support (Cu) of the sensor. After, THF was allowed to evaporate at room temperature (48 h); the membrane of the sensor (0.5 mm thick) was formed. Prior to EMF measurements, all the sensors were conditioned for 48h by soaking in 0.01M NaCl. The measurements were carried out at room temperature with a Mettler Toledo pH/ion analyzer by setting up the following cell:

Conductive support (Cu)/PVC membrane/test solution/Hg, Hg_2_Cl_2_, KCl (sat)

Potentiometric selectivity coefficients were determined according to the separate solution method by using the experimental EMF values obtained for 0.01 M of the tested cations and a theoretical slope of 59.2 mV/decade of activity for the primary cation (silver). The detection limit of each sensor was established at the point of intersection of the extrapolated linear mid-range and final low concentration level segments of the calibration plot.

### 4.3. Sample extraction for chloride determination

Meat product (10 g), previously homogenized, was weight and transferred into a 200 mL volumetric flask with water (100 mL). The solution was stirred and heated (80^°^C) for 30 minutes. After cooling, 2 mL of each Carrez reagent were added under stirring, allowed to settle (30 minutes) and then diluted to volume with deionized water. Finally, the solution was shaken and then filtered through filter paper into a 200 cm^3^ Erlenmeyer flask clean and dried. Extracted sample solution (20 mL) was used for each type of meat product in the potentiometric titration with silver nitrate 0.1 N (F = 1.00). The same volume of extracted solution was treated with excess of silver nitrate 0.1 N, and the residual silver nitrate was determined by titration with 0.1 N thiocyanate solution in the presence of nitrobenzene (Volhard method).

## Figures and Tables

**Table 1.** Composition of PVC membranes of (**A**) and (**B**) and response characteristics of Ag^+^ selective electrodes based on them.

**Sensor**	**Ionophore (I)**	**Composition of membranes (w/w)**						**Working conc. Range (M)**	**Slope (mV/decade)**
		**I**	**PVC**	**Add**	**DOS**	**DOP**	**NPOE**		
**1**	**A**	5	165	2	-	330	-	5×10^-5^ − 1×10^-1^	31.5
**2**		5	165	2	330	-	-	1×10^-5^ − 1×10^-1^	26.9
**3**		5	165	2	-	-	330	1×10^-5^ − 5×10^-2^	32.2
**4**		10	165	5	330	-	-	1×10^-5^ − 1×10^-1^	49.7
**5**		10	165	5	-	-	330	1×10^-5^ − 1×10^-1^	57.9
**6**		5	150	2	200	-	-	5×10^-4^ − 1×10^-1^	35.4
**7**	**B**	10	165	5	-	330	-	3×10^-5^ − 1×10^-1^	61.9
**8**		10	165	5	330	-	-	8×10^-6^ −1×10^-1^	64.5
**9**		10	165	5	-	-	330	1×10^-5^ − 1×10^-1^	55.8

**Table 2.** Chloride (g/kg) in different meat products determined by potentiometric titration (using sensor no. 8) and Volhard titration.

**Meat product**	**Potentiometric titration**	**Volhard titration**

Pate	12,3 ± 0,1	12,4 ± 0,1
Parizer	13,2 ± 0,2	13,1 ± 0,3
Salami	13,5 ± 0,1	13,6 ± 0,2
Sausage	15,9 ± 0,1	16,0 ± 0,2

**Table 3.** Determination of lead in synthetic and real samples by potentiometry (using sensor no. 9) and by AAS.

**Sample**	**Potentiometric μg/ml ± SD (n = 4)**	**AAS μg/ml ± SD (n = 4)**	**Recovery %**
1*	11.1 ± 0.3	10.3 ± 0.2	99.1
2*	31.6 ± 0.4	30.9 ± 0.3	101.6
3*	41.7 ± 0.3	41.0 ± 0.2	100.6
wastewater	30.1 ± 0.4	29.8 ± 0.2	

* synthetic samples

**Table 4.** Determination of copper in synthetic samples.

**Method**	* **mg/mL Cu^2+^**	**Recovery %**
Direct potentiometry	3.24 ± 0.3	96.1
Standard addition potentiometry	3.23 ± 0.4	95.8
AAS	3.33 ± 0.2	98.8

* Sample content: 3.37 mg/ml Cu^2+^

Average of four measurements
